# Electromyography of the Multifidus Muscle in Horses Trotting During Therapeutic Exercises

**DOI:** 10.3389/fvets.2022.844776

**Published:** 2022-05-27

**Authors:** Tena Ursini, Karen Shaw, David Levine, Jim Richards, Henry Steve Adair

**Affiliations:** ^1^Department of Large Animal Clinical Sciences, College of Veterinary Medicine, Equine Performance and Rehabilitation Center, University of Tennessee, Knoxville, TN, United States; ^2^Department of Physical Therapy, University of Tennessee at Chattanooga, Chattanooga, TN, United States; ^3^Allied Health Research Unit, University of Central Lancashire, Lancashire, United Kingdom

**Keywords:** electromyography, multifidus, rehabilitation, back pain, equine

## Abstract

Thoracolumbar pain has been identified in both human and equine patients. Rehabilitation and conditioning programs have focused specifically on improving trunk and abdominal muscle function (1–5). Equine exercise programs routinely incorporate ground poles and training devices for the similar goals of increasing spinal and core stability and strength (6–8). The multifidus muscle has been an area of focus due to atrophy associated with disease (9). To date, there have been no reports on the activity of the multifidus muscle in horses in relation to therapeutic exercises. Our objectives were to use electromyography to determine the average work performed and peak muscle activity of the multifidus in horses trotting, trotting over ground poles, trotting while wearing a resistance band-based training device and trotting while wearing the training device over ground poles. We hypothesized that ground poles and the training device would each increase average work performed and peak multifidus muscle activity. Right and left cranial thoracic locations showed significant increased muscle work and peak activation when horses were trotted over ground poles versus without. The peak activation was significantly greater in horses trotting over poles in both lumbar regions, but there was no significant change in peak activation in either location due to the training device. When the influence of the training device was investigated without ground poles, left caudal thoracic muscle work and peak activity, and right lumbar muscle work were significantly lower when using the training device, as compared to without. When the training device was combined with trotting over ground poles, both left and right caudal thoracic regions showed significantly lower muscle work and peak activity when the device was used. There was no significant difference between with and without the device in either left or right lumbar muscle work. In conclusion, implementing ground poles can be an effective strategy to increase the activation of the multifidus muscle, however, caution should be taken when incorporating the use of a resistance band training device as muscle work and peak activation were significantly reduced in most locations. Further study should be performed in regards to the training device to determine its effects on epaxial musculature.

## Introduction

In humans, paraspinal musculature has been shown to contribute a substantial portion of overall spinal stability (10, 11). The multifidus muscle has been specifically identified as a major contributor to spinal stabilization in humans (12). Spinal instability has been correlated to injury, even under low stress movements of daily living (13). Additionally, it has been hypothesized that a build-up of microtrauma could induce changes in neuromuscular control, thus predisposing spinal components to further injury (14).

Lower back pain (LBP) is defined as the pain of the posterior trunk between the 12th rib and the lower gluteal folds (15). A myriad of underlying conditions can cause LBP including but not limited to intervertebral disc herniation, spinal stenosis, degenerative scoliosis, osteoarthritis of the facet joints, and idiopathic causes (16, 17). While horses can have similar symptoms of LBP as seen in humans, the underlying cause is not always as clear. Veterinary clinicians are limited in their ability to diagnose specific spinal lesions in horses due to their size and the difficulty to perform advanced diagnostic imaging. Regardless of the cause of LBP in humans, treatment relies heavily on physical therapy to improve trunk and abdominal muscle function (1–5), as well as proprioception and balance (1, 3, 18). Similar principles have been implemented into equine therapeutic exercise programs with the use of ground poles and other training devices.

Ground poles are routinely used in equine exercise programs to improve proprioception, increase stride length, promote symmetry, and induce joint flexion (6, 7). Brown et al. has shown horses trotting over ground poles successfully clear the obstacle by lifting their limbs higher and increasing joint flexion across all joints (19). There was significantly more joint flexion when trotting over poles as compared to flat ground (19). It was concluded that trotting over poles would be effective to increase activation and strength of flexor muscles. During the stance phase, horses did not show significant increases in vertical ground reaction force or extension of the metacarpophalangeal and metatarsophalangeal joints (20). Thus, the load placed upon each limb was like that traveling across flat ground (20). To date, muscle activity has not been directly reported in horses trotting over ground poles.

Several types of training devices have been developed and used in equine exercise programs. Overall, the intention of these devices is to promote abdominal lifting, engagement of the hind limbs, and spinal stability while strengthening the epaxial musculature (21). One resistance band training device was determined to reduce mediolateral and rotational motion of the thoracolumbar spine (8). The authors concluded that this decrease in thoracolumbar motion was due to increased dynamic stability (8). If human modeling data is extrapolated, this would likely be due to increased muscle activity, since muscles contribute a large part to spinal stability (10, 11). Muscle activity was not assessed in the aforementioned resistance band-based device (8). Cottrail et al. described the activity of the longissimus dorsi muscle while using a different training device (21). The longissimus dorsi muscle is a large epaxial muscle in horses thought to contribute to dynamic spinal stability (22). Cottrall et al. did not find any significant increase in longissimus dorsi activation with the use of the training device (21). Therefore, if either of these training aids improve dynamic spinal stability, another mechanism or muscle is likely to be involved.

Electromyography (EMG) is the study of muscle activity by assessing the action potentials created by the motor unit (23). The activity of deep musculature can be recorded using in-dwelling fine wire electrodes without the potential for cross-talk from other muscles (23). The multifidus muscle can be imaged with routine ultrasonography (9, 24) in order to direct accurate and precise electrode placement.

Our objectives were to use electromyography to determine the average activation performed and peak muscle activity of the multifidus in horses trotting over ground poles and while wearing a resistance band-based training device. We hypothesized that ground poles and the training device would each increase average activation performed and peak multifidus muscle activity.

## Materials and Methods

### Horses

Four horses from the University of Tennessee Veterinary Research and Teaching herd were included. Any horse with greater than a grade 2 lameness based on the American Association of Equine Practitioners lameness scale were excluded. Gaited horses and gaited breeds were excluded unless they maintained a consistent diagonal two beat trot gait. This study was performed in accordance of the Assessment and Accreditation of Laboratory Animal Care and United States Department of Agriculture guidelines with approval from the University of Tennessee Institutional Animal Care and Use Committee.

### Gait Cycle Validation

The gait cycle was linked to the activity of the longissimus dorsi muscle. Self-adhesive surface electrodes with an inter-electrode distance of 2 cm were adhered to clipped, shaved, and cleaned skin overlying the longissimus dorsi muscle at the level of the dorsal spinous process of the 16th vertebrae as previously described (22).

In addition to having surface EMG sensors in place, 9 mm spherical reflective markers were placed on the lateral aspect of each hoof at the level of the coronary band. Using motion analysis (Nexus, Vicon Motion Systems, Oxford, England) integrated and synchronized with the electromyographic signal from a telemetric system (Myomotion; Noraxon USA, Scottsdale, USA), the timing of the longissimus dorsi muscle activity in relation to the gait cycle was determined.

Kinematic data from both motion capture cameras and electromyography were collected using Nexus software and imported into Visual3D (C-Motion Inc., Germantown MD, USA) for further processing. Kinematic data were interpolated and low-pass filtered with a frequency cut off of 8 Hz. Gait cycle events of heel strike and toe off of each hoof were labeled based on when makers reached a zero position in the vertical *z*-plane.

### Fine-Wire Electromyography

**Figure 1:**
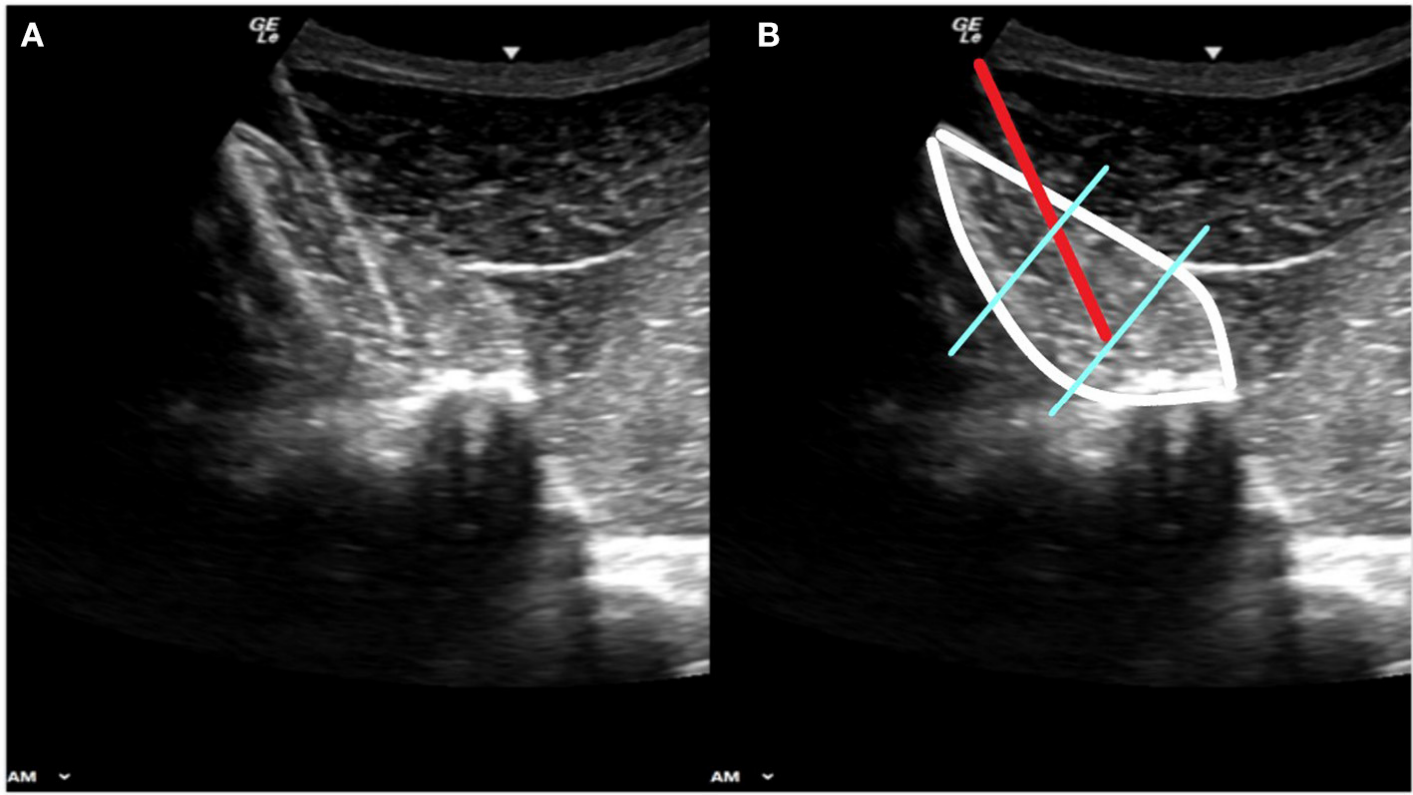
Panels **(A)** and **(B)** show the same diagnostic ultrasound image. Panel **(B)** shows the outline of the multifidus muscle (white border) the 23 gauge needle carrying the fine wire electrodes (red line) with the electrode ends embedded at the junction of the middle and deep thirds of the muscle belly (blue lines).

Muscle potentials from the multifidus muscle were collected using a telemetric unit (Myomotion; Noraxon USA, Scottsdale, AZ) with a sampling frequency of 1,500 Hz. The skin was clipped, shaved, and cleaned using chlorhexidine and isopropyl alcohol. Ultrasound was used to locate and identify each dorsal spinous process. The skin was desensitized with 1 ml mepivacaine per site taking care to remain superficial to the thoracolumbar fascia to prevent alterations in thoracolumbar muscle function as previously reported (18). Briefly, 23 gauge 75 mm length needles with pre-loaded paired electrodes (Chalgren Enterprises, Gilroy, CA) were aseptically inserted through the skin and visualized with ultrasound guidance to into the multifidus at the junction of the middle and deep third (Figure 1). The needles were removed, and the hook ended electrodes remained embedded in the muscle. No redirection of the needles was allowed given the potential for damaging the electrode ends. If the intended location was not achieved with the first insertion, the needle was removed and a new pre-loaded needle was used. Electrodes were placed at the level of the dorsal spinous process of the twelfth (T12) and eighteenth thoracic (T18) and fifth lumbar (L5) vertebrae bilaterally. Wires were connected to the EMG sensors using a screw post and nut device (DTS Fine wire lead connector, Noraxon USA, Scottsdale, AZ).

### Exercises

Electromyography signals were collected with the horse traveling straight in hand on synthetic arena footing under four separate conditions: trotting over a series of ground poles 10 cm in diameter, while wearing a therapeutic band-based training device (Equicore Concepts, East Lansing MI), trotting over the ground poles while also wearing the training device, and trotting over the same arena surface without either ground poles or therapeutic band exercise device. Distance between poles was approximately one meter, dependent upon the height and natural stride length of each individual horse. Horses were acclimated to the resistance band training device for a minimum of 3 days before data collection. Tension of each of the resistance bands was set to 25% (the length of the elastic resistance band was made to be 75% of the measured distance between the attachment points). The authors find this degree of tension most clinically effective and is comparable to other studies (8). The head and neck were maintained in a neutral position for every exercise. Video recording was synchronized to the telemetric system (Ninox Video Capture 125), to confirm the quality of each exercise. Horses had to perform between six and 15 consecutive and consistent strides for each exercise to be deemed a quality repetition. A minimum of five quality repetitions of each exercise were recorded. All horses had complete data for all multifidus locations. However, the T12 electrodes had to be removed before equipping the training device, resulting in comparisons only at T18 and L5 for the resistance device.

### Exercise Data Processing

**Figure 2:**
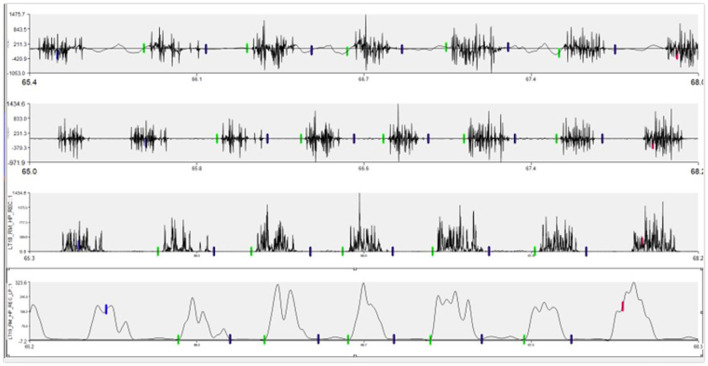
Example of EMG signal changes through processing process. Top row is the raw signal as collected. Second row contains the signal after a high pass filter of 40 Hz was applied. The third row represents rectification. The bottom row is the final enveloped signal after the 15 Hz low pass filter.

Motion artifact and noise from raw EMG signals was removed with a high-pass filter set at 40 Hz. Whole signals were then rectified. Lastly, a low pass filter was implemented with a 15 Hz cut off frequency. Using enveloped data, the onset and offset of muscle electrical activity within each of the five three-stride sections was labeled using Visual3D. Each of these activations were exported from Visual3D from the rectified and enveloped signals. The average rectified value and the maximum enveloped value were normalized to the maximal reference voluntary contraction, represented by the maximum EMG outcome measure observed across all trot strides for each horse, as previously described (25, 26). The average rectified signal (ARV) during the activation was used as an indication of average “work-done” by the muscle (27, 28). The peak value (PE) observed from the enveloped data represented the highest level of activation (27, 28) (Figure 2).

### Statistical Analysis

Statistical analysis of the data was performed using SPSS Version 27. The statistical analysis of the EMG measures included all observations across the two factors; with and without the training device and with and without ground poles. A two-factor univariate analysis of variance was used to test for differences between the two factors across all observations. Any interactions between the factors were further explored with unpaired *t*-tests.

## Results

### Horses

One gelding and three mares aged 4 to 14 years of various breeds from the University of Tennessee Veterinary Research and Teaching herd were utilized. All horses were deemed to be a grade 2 or less baseline lameness in any limb based on the American Association of Equine Practitioners lameness scale. All horses received oral phenylbutazone at a dose of 2.2 mg/kg twice daily started at least 24 h before data collection. All horses were visually sound during data collection as deemed by two experienced lameness veterinarians.

### Gait Cycle Validation

The left longissimus muscle was determined to have two isolated peaks of activation per single trotting gait cycle. The first peak was associated with left front toe off, and the second peak was associated with left hind toe off, consistent with previously reported work (22). Using the data collected from the left longissimus muscle, the timing of three complete gait cycles was determined and extrapolated to the synchronized signal of the sensors implanted within multifidus muscle. Five three-stride segments were isolated from the data sets previously confirmed to be a quality repetition based on the video recording.

### Fine-Wire Electromyography

Right and left T12 locations showed significant increases in bothARV and PE when horses were trotted over ground poles versus without (*p* < 0.001; Table 1).

**Table 1:** Means (standard deviation) and comparisons of normalized EMG outcome measures for all conditions.

		**Mean (SD)**				**Poles vs. no poles**		**Training device vs. no training device**		
**Muscle**	**Outcome measure**	**No poles, no training device**	**Poles, no training device**	**Training device, no poles**	**Training device, poles**	**Mean difference (±95% CI)**	***p*-Value**	**Mean difference (±95% CI)**	***p*-Value**	**Interaction**
Left T12	Average rectified	0.4434 (0.23556)	0.6179 (0.26349)	n/a	n/a	0.175	<0.001 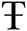			
	Peak envelope	0.5057 (0.26889)	0.7236 (.26351)	n/a	n/a	0.218	<0.001 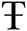			
Left T18	Average rectified	0.4391 (0.28076)	0.5281 (0.30866)	0.3472 (0.17994)	0.2728 (0.16772)	0.007	0.756	**–**0.174	<0.001	<0.001*
	Peak envelope	0.5224 (0.32428)	0.6031 (0.33821)	0.4016 (0.20246)	0.3521 (0.18241)	0.016	0.556	**–**0.186	<0.001	0.014*
Left L5	Average rectified	0.2715 (0.26597)	0.4090 (0.27310)	0.3710 (0.22303)	0.4334 (0.25320)	0.1	<0.001	0.062	<0.001	0.005*
	Peak envelope	0.2308 (0.26436)	0.3821 (0.32181)	0.2897 (0.15635)	0.3657 (0.23664)	0.114	<0.001 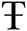	0.021	0.373	0.114
Right T12	Average rectified	0.5869 (0.40726)	0.8426 (0.28228)	n/a	n/a	0.256	<0.001 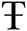			
	Peak envelope	0.6567 (0.43235)	0.9611 (0.35881)	n/a	n/a	0.304	<0.001 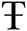			
Right T18	Average rectified	0.3049 (0.23703)	0.3866 (0.32605)	0.2687 (0.19079)	0.2618 (0.15112)	0.037	0.09	**–**0.081	<0.001	0.045*
	Peak envelope	0.3775 (0.26039)	0.4403 (0.35443)	0.3421 (0.22136)	0.3344 (0.19004)	0.028	0.264	**–**0.071	0.004	0.004*
Right L5	Average rectified	0.1833 (0.15608)	0.2347 (0.19312)	0.1670 (0.11342)	0.2006 (0.15582)	0.042	0.004	**–**0.025	0.087	0.001*
	Peak envelope	0.1489 (0.12945)	0.1801 (0.14016)	0.1441 (0.09891)	0.1789 (0.18250)	0.033	0.011 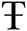	**–**0.003	0.817	0.887

^*^Significant interaction, conclusions were based on further post-hoc testing.

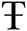
*Significance (p < 0.05)*.

When considering the multifidus locations tested both with and without the training device and with and without ground poles, significant interactions were seen between the two in all but the PE for left and right L5. The PE for both right (*p* < 0.011) and left (*p* < 0.001) L5 was significantly greater in horses trotting over poles vs. no poles, but there was no significant change in PE in either location due to the training device (Table 1).

For the locations that showed significant interactions between the conditions, *post hoc* unpaired t-tests, were used to compare with and without the training device in the with and without ground poles conditions separately.

When the influence of the training device was investigated without ground poles, left T18 ARV (*p* = 0.002) and PE (*p* < 0.001) and right L5 ARV (*p* < 0.001) were significantly lower when using the training device, as compared to without the training device (Table 2).

**Table 2:** *Post hoc* evaluation of training device without ground poles.

**Muscle**	**Outcome measure**	**No training device Mean (SD)**	**Training device Mean (SD)**	***p*-Value for equality of means (2-tailed)**	**Mean difference**	**95% CI (lower)**	**95% CI (upper)**
Left T18	Average rectified	0.4647 (0.31870)	0.3472 (0.17994)	0.002 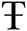	0.11745	0.04516	0.18975
	Peak envelope	0.5717 (0.38510)	0.4016 (0.20246)	<0.001 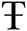	0.1701	0.08413	0.25607
Left L5	Average rectified	0.3608 (0.28953)	0.3710 (0.22303)	0.78	**–** 0.0102	**–** 0.08227	0.06187
Right T18	Average rectified	0.2940 (0.26092)	0.2687 (0.19079)	0.435	0.02528	**–** 0.0385	0.08906
	Peak envelope	0.3887 (0.29459)	0.3421 (0.22136)	0.208	0.04658	**–** 0.02612	0.11929
Right L5	Average rectified	0.2435 (0.15558)	0.1670 (0.11342)	<0.001 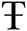	0.07647	0.03848	0.11446

*
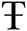
Significance (p < 0.05)*.

When the training device was combined with trotting over ground poles, both left T18 PE (*p* < 0.001) and ARV (*p* < 0.001) and right T18 PE (*p* < 0.001) and ARV (*p* < 0.009) were significantly lower when the device was used. There was no significant difference between with and without the device in either left or right L5 ARV (Table 3).

**Table 3:** *Post hoc* evaluation of training device with ground poles.

**Muscle**	**Outcome measure**	**No training device mean (SD)**	**Training device mean (SD)**	***p*-Value for equality of means (2-tailed)**	**Mean difference**	**95% CI (lower)**	**95% CI (upper)**
Left T18	Average rectified	0.5281 (0.30866)	0.2728 (0.16772)	<0.001 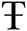	0.25529	0.18589	0.32469
	Peak envelope	0.6031 (0.33821)	0.3521 (0.18241)	<0.001 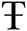	0.25098	0.17506	0.32689
Left L5	Average rectified	0.4090 (0.27310)	0.4334 (0.25320)	0.514	**–** 0.02434	**–** 0.09778	0.0491
Right T18	Average rectified	0.3866 (0.32605)	0.2618 (0.15112)	<0.001 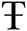	0.1248	0.05375	0.19585
	Peak envelope	0.4403 (0.35443)	0.3344 (0.19004)	0.009 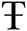	0.10588	0.02642	0.18533
Right L5	Average rectified	0.2347 (0.19312)	0.2006 (0.15582)	0.171	0.03408	**–** 0.01487	0.08302

*
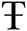
Significance (p < 0.05)*.

The clinical importance of muscle activation for each exercise and location were also calculated as a percentage of change as compared to the baseline condition of trotting over flat ground (Table 4). Ground poles cause a general increase in both PE and ARV at all locations. The highest magnitude of change was seen in both T12 locations with increases of approximately 40%−50% in both ARV and PE. Left L5 exhibited increases in ARV and PE of 51 and 66% respectively. Left and right T18, and right L5 showed increases of 15%−30%. The training device caused decreases in both ARV and PE in all locations except left L5. Of note were decreases of 21 and 23% in ARV and PE respectively at left T18. When the training device and ground poles were used in combination, larger decreases in ARV and PE were observed at left and right T18 locations. Left and right L5 both showed effects similar to that was seen with ground poles alone (Table 4).

**Table 4:** Percent change in outcome measure means for each exercise condition in comparison to baseline.

**Muscle**	**Outcome measure**	**No poles, no training device mean (baseline)**	**Poles no training device mean**	**% change 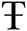 **	**No poles training device mean**	**% change 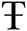 **	**Poles and training device mean**	**% change 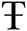 **
Left T12	Average rectified	0.4434	0.6179	39%				
	Peak envelope	0.5057	0.7236	43%				
Left T18	Average rectified	0.4391	0.5281	20%	0.3472	**–**21%	0.2728	**–**38%
	Peak envelope	0.5224	0.6031	15%	0.4016	**–**23%	0.3521	**–**33%
Left L5	Average rectified	0.2715	0.409	51%	0.371	37%	0.4334	60%
	Peak envelope	0.2308	0.3821	66%	0.2897	26%	0.3657	58%
Right T12	Average rectified	0.5869	0.8426	44%				
	Peak envelope	0.6567	0.9611	46%				
Right T18	Average rectified	0.3049	0.3866	27%	0.2687	**–**12%	0.2618	**–**14%
	Peak envelope	0.3775	0.4403	17%	0.3421	**–**9%	0.3344	**–**11%
Right L5	Average rectified	0.1833	0.2347	28%	0.167	**–**9%	0.2006	9%
	Peak envelope	0.1489	0.1801	21%	0.1441	**–**3%	0.1789	20%

*
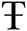
Positive value indicates an increase in mean muscle activity of that condition as compared to the baseline condition of trotting without either the ground poles or training device. A negative value indicates a decrease in mean muscle activity*.

## Discussion

The multifidus muscle has garnered much attention in the equine literature due to implied associations of atrophy with axial spine disease (9) like what is reported in humans (29–34). Rehabilitation methods have focused on promoting hypertrophy of this structure (24, 35) however, muscle activity has never been directly measured. The work presented here is the first to document the overall muscle work and peak activity of the multifidus muscle in relation to specific therapeutic exercises and training devices.

Other back and hind limb muscles have been successfully investigated in the horse using electromyography (21–23, 25, 36–39). The longissimus dorsi muscle is noted to produce two bursts of activity for each trot stride with the main function of the longissimus dorsi suspected to provide overall spinal stiffness specifically in the sagittal plane (22, 38). The multifidus muscle is speculated to have a similar function, however the fasiculated anatomy indicate it may be more suited to provide minute and rapid intersegmental stabilization. The activity of the multifidus has yet to be related to spinal motion in horses. The longissimus dorsi activation pattern has been noted to be increasingly variable in lame horses (36). It is unknown if the multifidus is similarly affected by the asymmetric motion associated with hind limb lameness.

We hypothesized that having horses trot over poles would increase the average muscle activation and peak activity of the multifidus as compared to trotting over the same surface without poles. This work supported that hypothesis in that both cranial thoracic regions showed significant increases in ARV and PE. Additionally, trotting over ground poles induced significantly more PE in left and right L5. Ground poles increased the ARV by 20%−51% in comparison to trotting over the same surface without poles in all locations. Similarly, the PE increased by 15%−66% across all multifidi locations measured.

We also hypothesized that when horses exercised wearing a resistance band-based training device the average and peak muscle activity would increase. Our findings did not support this hypothesis and actually resulted in significantly less ARV and PE in several locations. Other locations showed no significant change in ARV or PE when the device was used as compared to without it. Interestingly, the mean of each outcome parameter and muscle location except the ARV of left L5 was lower when the training device was used as compared to the same conditions without it. With a larger sample size, more locations may have reached statistical significance.

When the clinical effects were calculated based on a percentage of the baseline condition, each of the T18 locations showed the largest decrease in muscle activation when ground poles were used in conjunction with the training device. The L5 locations each had results lower, but more similar to that of horses trotting over ground poles without the device. Therefore, the use of both ground poles and the training device promoted further decrease in activity in the caudal thoracic regions, and maintained a similar muscle output as if the device was not used in the lumbar areas.

The overall decrease in average and peak muscle activity seen with the use of the training device was surprising. Clinically, horses do seem to engage their back and hindquarters when the device is used. Pfau et al. found that horses who were exercised in the training device had decreased roll, pitch, and mediolateral displacement of the thoracolumbar region (8). They concluded the resistance band training device increased dynamic stability. However, our work implies that the decrease in motion is not due to increased multifidus activity. It is possible that the use of the training device activates other spinal stabilizers or abdominal or hind limb muscles. Similar studies have investigated the effects of a training device on the longissimus dorsi muscle, the main contributor of the epaxial muscle group in horses (21). They discovered that the training device also significantly decreased the muscle activity (8). Similar reductions in longissimus dorsi activity have been seen with the resistance band training device (25). The training device may alter the timing of activation and while the overall muscle work or peak activation were unchanged, the muscle may be active during a different phase of stride, providing more stability during motion. To more precisely determine the function of the multifidus muscle during motion, more advanced motion analysis should be performed in conjunction with multifidus EMG recording. Additionally, the training device may require a more prolonged training regimen to change muscle activation.

Specific limitations of this work include the inability to make conclusions based on the timing of the multifidus muscle activation in reference to each phase of the stride. This was not a primary objective of this study, as we were interested in the overall muscle activity due to therapeutic interventions, not classifying the timing of contractions. As stated previously, the multifidus muscle has several fascicles of varying lengths (40, 41). We took exceptional care to implant each sensor at a similar location and depth. However, the fascicles are not distinguishable on ultrasound, and therefore, some electrodes may be in different fascicles than others. While the anatomy is well documented (40, 41), the function of each fascicle has not yet been determined. Hyytiainen et al. (42) has shown variation of muscle fiber types between fascicles in horses as well as breeds. Muscles have been documented to alter in fiber type, based on the forces and functions required (43). Thus, there could be variation in EMG activity between fascicles. This work incorporates the use of four horses. Given the strongly significant results in some locations, we felt a sample size of four was adequate to explore the immediate effects of the conditions tested. Additionally, using all observations resulted in a calculated power of 1 at each muscle location and outcome measure. However, more changes could become evident with more horses. Lastly, velocity could not be standardized between trials, however, horses were kept at their own natural pace for each exercise repetition and care was taken to prevent fatigue. This is similar to other methods used (25, 26, 39). Additionally, each horse was maneuvered by the same handler throughout the study period, thus limiting the effect of variation from different handlers.

In conclusion, ground poles should be incorporated into every reconditioning and exercise plan focused on activating the multifidus muscle. However, caution should be used in regards to the resistance band training device tested, as both average and peak muscle activation were significantly lower in several locations. Further work should be performed to investigate the effects of the training device on other spinal stabilizing epaxial musculature and in conjunction with motion analysis.

## Data Availability Statement

The original contributions presented in the study are included in the article/supplementary material, further inquiries can be directed to the corresponding author.

## Ethics Statement

The animal study was reviewed and approved by University of Tennessee Institutional Animal Care and Use Committee.

## Author Contributions

TU performed all data collection, processing, and main manuscript preparation. KS assisted with data collection and manuscript editing. DL and JR assisted with study design, statistical analysis, data processing, and manuscript editing. HA assisted with study design, and manuscript editing. All authors contributed to the article and approved the submitted version.

## Funding

Funding for this project was provided by the Large Animal Clinical Sciences Department at the University of Tennessee.

## Conflict of Interest

The authors declare that the research was conducted in the absence of any commercial or financial relationships that could be construed as a potential conflict of interest.

## Publisher's Note

All claims expressed in this article are solely those of the authors and do not necessarily represent those of their affiliated organizations, or those of the publisher, the editors and the reviewers. Any product that may be evaluated in this article, or claim that may be made by its manufacturer, is not guaranteed or endorsed by the publisher.

## References

[1] Areeudomwong P Buttagat V. Comparison of core stabilisation exercise and proprioceptive neuromuscular facilitation training on pain-related and neuromuscular response outcomes for chronic low back pain: a randomised controlled trial. Malaysian J Med Sci. (2019) 26:77–89. 10.21315/mjms2019.26.6.8 31908589 PMC6939725

[2] Berry DB Padwal J Johnson S Englund EK Ward SR Shahidi B. The effect of high-intensity resistance exercise on lumbar musculature in patients with low back pain: a preliminary study. BMC Musculoskeletal Disord. (2019) 20:290. 10.1186/s12891-019-2658-1 31208400 PMC6580468

[3] Farragher JB Pranata A Williams G El-Ansary D Parry SM Kasza J. Effects of lumbar extensor muscle strengthening and neuromuscular control retraining on disability in patients with chronic low back pain: a protocol for a randomised controlled trial. BMJ Open. (2019) 9:e028259. 10.1136/bmjopen-2018-028259 31431445 PMC6707707

[4] Tagliaferri SD Miller CT Ford JJ Hahne AJ Main LC Rantalainen T. Randomized trial of general strength and conditioning versus motor control and manual therapy for chronic low back pain on physical and self-report outcomes. J Clin Med. (2020) 9:1726. 10.3390/jcm9061726 32503243 PMC7355598

[5] Wirth K Hartmann H Mickel C Szilvas E Keiner M Sander A. Core stability in athletes: a critical analysis of current guidelines. Sports Med. (2017) 47:401–14. 10.1007/s40279-016-0597-7 27475953

[6] Frick A Hethcote B. Fitness in Motion: Keeping Your Equine's Zones at Peak Performance. G - Reference, Information and Interdisciplinary Subjects Series. Guilford, CT: Globe Pequot Press (2007).

[7] McGowan CM Goff L. Animal Physiotherapy: Assessment, Treatment, and Rehabilitation of Animals, 2^nd^ ed. Chichester: John Wiley & Sons Inc (2016).

[8] Pfau T Simons V Rombach N Stubbs N Weller R. Effect of a 4-week elastic resistance band training regimen on back kinematics in horses trotting in-hand and on the lunge. Equine Vet J. (2017) 49:829–35. 10.1111/evj.12690 28432739

[9] Stubbs NC Riggs CM Hodges PW Jeffcott LB Hodgson DR Clayton HM. Osseous spinal pathology and epaxial muscle ultrasonography in thoroughbred racehorses. Equine Vet J. (2010) 42:654–61. 10.1111/j.2042-3306.2010.00258.x 21059076

[10] Crisco JJ Panjabi MM. *Euler stability of the human ligamentous lumbar spine. 1*: theory. Clin Biomech. (1992) 7:19–26. 10.1016/0268-0033(92)90003-M 23915612

[11] Crisco JJ Panjabi MM Yamamoto I Oxland TR. Euler stability of the human ligamentous lumbar spine. 2: Experiment. Clin Biomech. (1992) 7:27–32. 10.1016/0268-0033(92)90004-N 23915613

[12] Barr KP Griggs M Cadby T. Lumbar stabilization - core concepts and current literature, part 1. Am J Phys Med Rehabil. (2005) 84:473–80. 10.1097/01.phm.0000163709.70471.42 15905663

[13] McGill SM. Low back stability: from formal description to issues for performance and rehabilitation. Exerc Sport Sci Rev. (2001) 29:26–31. 10.1097/00003677-200101000-00006 11210443

[14] Panjabi MM. A hypothesis of chronic back pain: ligament subfailure injuries lead to muscle control dysfunction. Eur Spine J. (2006) 15:668–76. 10.1007/s00586-005-0925-3 16047209 PMC3489327

[15] Kalichman L Carmeli E Been E. The association between imaging parameters of the paraspinal muscles, spinal degeneration, and low back pain. BioMed Res Int. (2017) 2017:2562957. 10.1155/2017/2562957 28409152 PMC5376928

[16] Creze M Soubeyrand M Timoh KN Gagey O. Organization of the fascia and aponeurosis in the lumbar paraspinal compartment. Surg Radiol Anat. (2018) 40:1231–42. 10.1007/s00276-018-2087-0 30171298

[17] He K Head J Mouchtouris N Hines K Shea P Schmidt R. The implications of paraspinal muscle atrophy in low back pain, thoracolumbar pathology, and clinical outcomes after spine surgery: a review of the literature. Global Spine J. (2020) 10:657–66. 10.1177/2192568219879087 32677568 PMC7359686

[18] Hides JA Murphy M Jang E Blackwell L Sexton M Sexton C. Predicting a beneficial response to motor control training in patients with low back pain: a longitudinal cohort study. Eur Spine J. (2019) 28:2462–9. 10.1007/s00586-019-06045-7 31254095

[19] Brown S Stubbs NC Kaiser LJ Lavagnino M Clayton HM. Swing phase kinematics of horses trotting over poles. Equine Vet J. (2015) 47:107–12. 10.1111/evj.12253 24593249

[20] Clayton HM Stubbs NC Lavagnino M. Stance phase kinematics and kinetics of horses trotting over poles. Equine Vet J. (2015) 47:113–8. 10.1111/evj.12251 24580416

[21] Cottriall S Ritruechai P Wakeling JM. The effects of training aids on the longissimus dorsi in the equine back. Comp Exerc Physiol. (2009) 5:111. 10.1017/S1478061509342346

[22] Licka TF Peham C Frey A. Electromyographic activity of the longissimus dorsi muscles in horses during trotting on a treadmill. Am J Vet Res. (2004) 65:155–8. 10.2460/ajvr.2004.65.155 14974571

[23] Williams JM. Electromyography in the horse: a useful technology? J Equine Vet Sci. (2018) 60(C):43–58.e2. 10.1016/j.jevs.2017.02.005

[24] Stubbs NC Kaiser LJ Hauptman J Clayton HM. Dynamic mobilisation exercises increase cross sectional area of musculus multifidus. Equine Vet J. (2011) 43:522–9. 10.1111/j.2042-3306.2010.00322.x 21496085

[25] Shaw K Ursini T Levine D Richards J Adair S. The effect of ground poles and elastic resistance bands on longissimus dorsi and rectus abdominus muscle activity during equine walk and trot. J Equine Vet Sci. (2021) 107:103772. 10.1016/j.jevs.2021.103772 34802619

[26] St. George L Roy SH Richards J Sinclair J Hobbs SJ. Surface EMG signal normalisation and filtering improves sensitivity of equine gait analysis. Comp Exerc Physiol. (2019) 15:173–185. 10.3920/CEP190028

[27] Richards J Selfe J Sinclair J May K Thomas G. The effect of different decline angles on the biomechanics of double limb squats and the implications to clinical and training practice. J Hum Kinet. (2016) 52:125–38. 10.1515/hukin-2015-0200 28149400 PMC5260524

[28] Richards J Thewlis D Selfe J Cunningham A Hayes C. A biomechanical investigation of a single-limb squat: implications for lower extremity rehabilitation exercise. J Athl Train. (2008) 43:477–82. 10.4085/1062-6050-43.5.477 18833310 PMC2547867

[29] Battié MCP Niemelainen RP Gibbons LEP Dhillon SMD. Is level- and side-specific multifidus asymmetry a marker for lumbar disc pathology? Spine J. (2012) 12:932–9. 10.1016/j.spinee.2012.08.020 23084154

[30] Cooley JR Walker BF Ardakani EM Kjaer P Jensen TS Hebert JJ. Relationships between paraspinal muscle morphology and neurocompressive conditions of the lumbar spine: a systematic review with meta-analysis. BMC Musculoskeletal Disord. (2018) 19:351. 10.1186/s12891-018-2266-5 30261870 PMC6161433

[31] Faur C Patrascu JM Haragus H Anglitoiu B. Correlation between multifidus fatty atrophy and lumbar disc degeneration in low back pain. BMC Musculoskeletal Disord. (2019) 20. 10.1186/s12891-019-2786-7 31488112 PMC6729014

[32] Fortin M Lazary A Varga PP Battie MC. Association between paraspinal muscle morphology, clinical symptoms and functional status in patients with lumbar spinal stenosis. Eur Spine J. (2017) 26:2543–51. 10.1007/s00586-017-5228-y 28748488

[33] Liu Y Liu Y Hai Y Liu T Guan L Chen X. Multifidus muscle fatty infiltration as an index of dysfunction in patients with single-segment degenerative lumbar spinal stenosis: a case-control study based on propensity score matching. J Clin Neurosci. (2020) 75:139–48. 10.1016/j.jocn.2020.03.001 32169364

[34] Shafaq N Suzuki A Matsumura A Terai H Toyoda H Yasuda H. Asymmetric degeneration of paravertebral muscles in patients with degenerative lumbar scoliosis. Spine. (2012) 37:1398–406. 10.1097/BRS.0b013e31824c767e 22322373

[35] Oliveira K Soutello RVG Fonseca R Lopes AM Santos PCS Santos JMF. Biometry by ultrasonography of the epaxial and pelvic musculature in equines trained with Pessoa's rein. Ciencia Rural. (2014) 44:2045–51. 10.1590/0103-8478cr20130604

[36] Zaneb H Kaufmann V Stanek C Peham C Licka TF. Quantitative differences in activities of back and pelvic limb muscles during walking and trotting between chronically lame and nonlame horses. Am J Vet Res. (2009) 70:1129–34. 10.2460/ajvr.70.9.1129 19719429

[37] Robert C Audigié F Valette JP Pourcelot P Denoix JM. Effects of treadmill speed on the mechanics of the back in the trotting saddlehorse. Equine Vet J. (2001) 33:154–9. 10.1111/j.2042-3306.2001.tb05380.x 11721558

[38] Robert C Valette JP Pourcelot P Audigié F Denoix JM. Effects of trotting speed on muscle activity and kinematics in saddlehorses. Equine Vet J. (2002) 34:295–301. 10.1111/j.2042-3306.2002.tb05436.x 12405704

[39] St George L Clayton HM Sinclair J Richards J Roy SH Hobbs SJ. Muscle function and kinematics during submaximal equine jumping: what can objective outcomes tell us about athletic performance indicators? Animals. (2021) 11:414. 10.3390/ani11020414 33562875 PMC7915507

[40] Garcia Lineiro JA Graziotti GH Rodriguez Menendez JM Rios CM Affricano NO Victorica CL. Structural and functional characteristics of the thoracolumbar multifidus muscle in horses. J Anat. (2017) 230:398–406. 10.1111/joa.12564 27861847 PMC5314397

[41] Stubbs NC Hodges PW Jeffcott LB Cowin G Hodgson DR McGowan CM. Functional anatomy of the caudal thoracolumbar and lumbosacral spine in the horse. Equine Vet J Suppl. (2006) 38:393–9. 10.1111/j.2042-3306.2006.tb05575.x 17402454

[42] Hyytiainen HK Mykkanen AK Hielm-Bjorkman AK Stubbs NC McGowan CM. Muscle fibre type distribution of the thoracolumbar and hindlimb regions of horses: relating fibre type and functional role. Acta Vet Scand. (2014) 56:8. 10.1186/1751-0147-56-8 24468115 PMC3922740

[43] Castejon-Riber C Riber C Dolores Rubio M Aguera E Munoz A. Objectives, principles, and methods of strength training for horses. J Equine Vet Sci. (2017) 56:93–103. 10.1016/j.jevs.2017.04.011

